# Characterization of porcine milk oligosaccharides over lactation between primiparous and multiparous female pigs

**DOI:** 10.1038/s41598-018-23025-x

**Published:** 2018-03-16

**Authors:** Jinhua Wei, Zhuo A. Wang, Bing Wang, Marefa Jahan, Zhongfu Wang, Peter C. Wynn, Yuguang Du

**Affiliations:** 1 State Key Laboratory of Biochemical Engineering, Institute of Process Engineering, Chinese Academy of Sciences, Beijing, 100190 P.R. China; 2 Key Laboratory of Biopharmaceutical Production & Formulation Engineering, PLA, Beijing, 100190 P.R. China; 3 Graham Centre for Agricultural Innovation, Charles Sturt University, Wagga Wagga, NSW 2650 Australia; 4 Key Laboratory of Resource Biology and Biotechnology in Western China, Ministry of Education, College of Life Sciences, Northwest University, Xi’an, 710069 P.R. China

## Abstract

Milk oligosaccharides (MOs) are complex carbohydrates with multifunctional health benefits for the neonate. Poor reproductive performance in primiparous gilts limits their productivity. Changes in the structure and abundance of porcine MO (PMOs) through lactation with parity remains unknown and may explain superior new-born growth in litters from multiparous sows relative to gilts. We report 55 PMOs structures, of which 25 are new (17 sialylated and 8 neutral). Their incidence in gilt and sow colostrum was almost identical (53 *vs*. 54), but not in transitional milk (48 *vs*. 53) nor mature milk (41 *vs*. 47). These PMOs including neutral-, sialyl- and fucosyl- MOs in colostrum were more abundant in the gilt than the sow, but always decreased during lactation. Structural diversity decreased, although fucosylated MO were conserved. In conclusion, high diversity and levels of MO in porcine milk is parity dependent. Given the similarity between porcine and human MO profiles, our findings may help define key roles for MOs as potential dietary additives to improve growth of neonates from first pregnancies in both human and sows.

## Introduction

Milk is the most critically important primary source of nutrition for humans and all new-born mammalian species because of its enrichment with proteins, lipids and carbohydrates. Milk oligosaccharides (MOs), both neutral (N-OS) and sialylated (S-OS), are essential constituents in all mammalian milks. The total concentration of MOs in human (HMOs) colostrum is ~20–25 g/L^1^ and ~5–20 g/L in mature milk^1,2^. Thus, the MOs are the third largest dietary component in milk, exceeded only by the level of lactose (**~**70 g/L) and lipid (~40 g/L)^3^. MOs mediate a myriad of important biological functions required for skeletal growth and maturation, development of the immune and neural organ systems, establishment of gut microbiota and protection against GI diseases in infants^3–5^.

Mass spectral analyses of mammalian MOs have revealed a plethora of differences in the chemical structures and relative abundance from different mammalian species^6–10^ with >200 distinct structures reported^7,8,11,12^. This remarkable diversity is hypothesized to provide the structural basis for their multiple biological functions. Based on the continuing improvement in the sensitivity of mass spectral analyses, it is anticipated that an even greater number of unidentified MOs structures, and their cognate biological functions, will be discovered^9,13,14^.

Consistent with the structural diversity in MOs from mammalian species is the observation that their core structures generally consist of the hexoses, D-glucose (Glc), D-galactose (Gal), N-acetylglucosamine (GlcNAc), L-fucose (Fuc; 6-deoxy-L-galactose) and the nine-carbon acidic sugar, N-acetylneuraminic acid (Neu5Ac; Sia)^6,15,16^. Uniquely, however, is the finding of the N-glycolylneuraminic acid (Neu5Gc) as a sialic acid in goat, bovine and several other mammalian MOs^17^. This “non-human” sialic acid” is not a natural constituent in HMOs because humans lack the ability to synthesize Neu5Gc resulting from a genetic mutation several million years ago in the CMP-Neu5Ac hydroxylase (CMAH) gene that converts Neu5Ac to Neu5Gc^15,16^.

MOs are divided into two general classes, neutral and sialylated (acidic)^18,19^. Based on this classification, acidic MOs contain one or more Sia, in addition to sulfate or phosphate substituents. The neutral MOs often contain fucose covalently linked to the core hexose oligosaccharide backbone^6,20,21^. Structural studies have revealed that HMOs contain a larger number of structures with greater structural diversity compared with other mammalian species^17^. Presently, more than *ca*.200 HMOs structures have been reported^7,8,11,12^. Compared to human milk, the concentration of MOs in most domestic mammalian species is 10 to 100-fold lower^6^, e.g. bovine milk oligosaccharides (BMO) are represented at concentrations 20 to 100-fold lower than found in human milk (~1 g/L in colostrum^10,22^, ~0.05 g/L in mature milk^3^).

Domestic pigs are recognized as a preferred animal model for monogastric nutritional studies based on the similarity of their physiology and anatomical features of their digestive system with that of the human infant^23,24^. The pig also has comparable nutritional requirements^23,24,25^. For these reasons, there has recently been an increased interest in the nutritional composition of porcine milk, yet most studies have focused primarily on the composition of proteins, lipids (gangliosides) and carbohydrates^14,26,27^. As a consequence, there is a dearth of information on PMOs, particularly in different parity of pregnancy during lactation, and the important role these MOs play in growth and development. A critical objective of our studies, therefore, has been to chemically identify and quantify structural changes in PMOs during lactation in pigs in their first and subsequent pregnancies using isotope-labelling associated LC-ESI-MS/MS. This highly sensitive, stable isotope method is well suited for the separation, structural identification and quantitative analysis of MOs from various sources^13,28,29^. We also try to explored the potential impact of these structural differences on their functional role for animal production and reproduction as well as the growth and development of offspring. Our aim has been achieved by carrying out a comprehensive structural study using LC-ESI-MS/MS to analyze the MOs from gilt and sow milk during the course of lactation.

## Results

### Structural characterization and quantification of porcine milk oligosaccharides (PMOs) during lactation by HPLC-ESI-MS/MS identification

**Figure 1:**
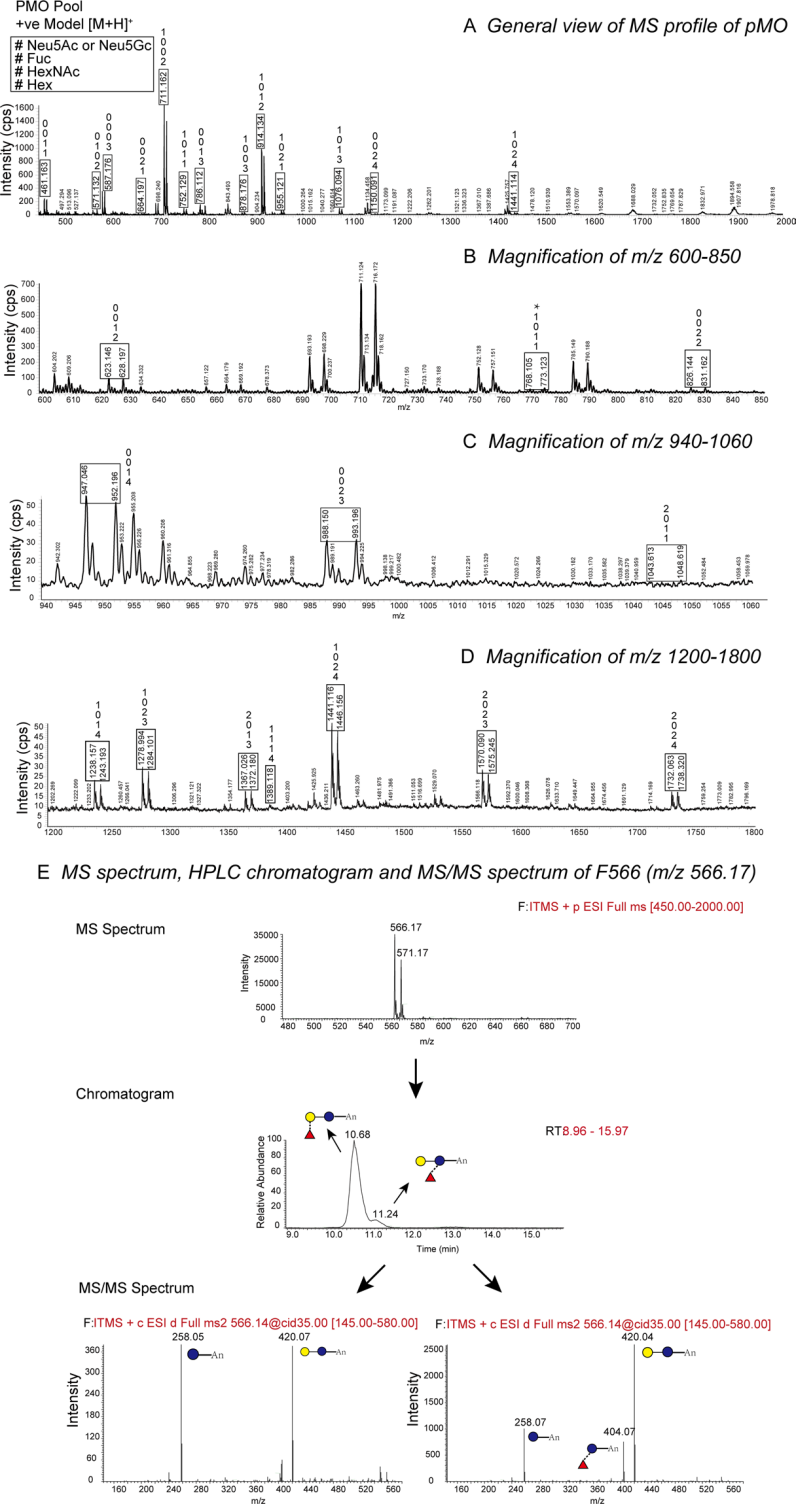
HPLC-ESI MS profile of a *d*_0_- and *d*_5_- aniline labeled PMOs pool in the positive-ion mode. (**A**) General view of MS profile of PMO; (**B**) Magnification of m/z 600~850; (**C**) Magnification of m/z 940~1060; (**D**) Magnification of m/z 1200~1800. The number peak represents, from bottom to top, the unit number of Hex, HexNAc, Fuc and Neu5Ac/Neu5Gc (black star) residues in a specific oligosaccharide. The vertical axis represents the signal intensity of the MS spectrum (counts per second, cps). (**E**) MS spectrum, HPLC chromatogram and MS/MS spectrum of F566 (m/z 566.17). Inserted diagram of oligosaccharide structures is based on the Oxford symbol nomenclature.

To facilitate annotation of PMO species in the mass spectral profiles, each milk sample was labelled with *d*_0_- and *d*_5_- aniline isotopes, respectively^30,31^. Labelled MOs samples were then mixed in a 1:1 ratio and analyzed by LC-ESI-MS/MS. The aniline labelled PMOs are shown in the mass spectra as two neighbouring isotopic peaks with 5 m/z difference (Fig. 1). Further structural analyses and annotation of the chemical structures of the PMOs were carried out by analysing the MS spectrum, HPLC chromatographic profile and the MS/MS spectrum for each candidate PMO peak observed in the mass spectra. Each sialic acid or neutral isomer was then annotated with the “GlycoWorkBench” software (version 2.1). The composition and proposed structures of the PMOs confirmed by both isotopic labelled twin peaks and MS/MS profile analyses during course of lactation are designated in Tables 1 and 2 as “known” and “unknown” PMO structures. Details are included in the Fig. 1 and supplementary information.

**Table 1:** Compositions and proposed structures of Porcine milk oligosaccharides (PMO) identified in this study----Known Structures.

No.	Designation^1^	m/z^2^ (expt)	m/z^2^ (cal)	Hex	HexNAc	Fuc	NeuAc	NeuGc	Retention Time (min)	Proposed Structures^3^	Colostrum		Transitional Milk		Mature Milk		Notes^4^
											Gilt	Sow	Gilt	Sow	Gilt	Sow	
**Acidic PMO**																	
1	S711-1	711.17	711.22	2			1		11.69		√	√	√	√	√	√	3′-SL
2	S711-2	711.17	711.22	2			1		13.44		√	√	√	√	√	√	6′-SL
3	S752-1*	752.22	752.25	1	1		1		11.38		√	√	√	√	√	√	3′/6′-SLN
4	S752-3*	752.22	752.25	1	1		1		15.99		√	√	√	√	√	√	3′/6′-SLN
5	S768	768.22	768.24	1	1			1	17.14		√	√	√	√	√	√	6′-Neu5GcLacNAc
6	S873-1	873.14	873.27	3			1		13.81		√	√	√	√	√	√	3′/6′S-3′/6′-GL
7	S873-2	873.14	873.27	3			1		14.61		√	√	√	√	√	√	3′/6′S-3′/6′-GL
8	S873-3	873.14	873.27	3			1		16.06		√	√	√	√	√	√	
9	S1043	1043.25	1043.34	1	1		2		17.11		√	√	—	√	—	√	DSLN
10	S1076-2	1076.12	1076.35	3	1		1		15.87		√	√	√	√	√	√	3′/6′-SLNnT
11	S1076-3	1076.12	1076.35	3	1		1		16.77		√	√	√	√	√	√	3′/6′-SLNnT
12	S1238-1*	1238.11	1238.40	4	1		1		18.74		√	√	√	√	√	√	3′/6′-SLNP I
13	S1238-2*	1238.11	1238.40	4	1		1		20.35		—	√	—	√	—	√	3′/6′-SLNP I
14	S1279-2	1279.06	1279.43	3	2		1		18.09		√	√	—	—	√	√	
15	SF1384*	1384.62	1384.46	4	1	1	1		10.13		√	√	√	√	—	—	
16	S1441-3*	1441.15	1441.48	4	2		1		22.24		√	√	√	√	—	—	SLNnH I/II
17	S1732-1*	1732.11	1732.58	4	2		2		21.40		√	√	√	√	—	√	DSLNnH
**Neutral PMO**																	
18	F566-1	566.15	566.18	2		1			10.93		√	√	√	√	√	√	2′-FL
19	F566-2	566.15	566.18	2		1			11.47		√	√	√	√	√	√	3′-FL
20	N582-1	582.17	582.18	3					12.09		√	√	√	√	√	√	Isoglobotriose/3′/6′-GL
21	N582-2	582.17	582.18	3					12.83		√	√	√	√	√	√	Isoglobotriose/3′/6′-GL
22	N582-3	582.17	582.18	3					13.35		√	√	√	√	√	√	Isoglobotriose/3′/6′-GL
23	N623-1*	623.15	623.20	2	1				11.81		√	√	√	√	√	√	
24	N623-2*	623.15	623.20	2	1				12.75		√	√	√	√	√	√	
25	N623-3*	623.15	623.20	2	1				13.40		√	√	√	√	√	√	
26	N785-1	785.15	785.26	3	1				14.53		√	√	√	√	√	√	
27	N826*	826.16	826.28	2	2				14.61		√	√	√	√	√	√	
28	N947-1*	947.14	947.31	4	1				18.03		√	√	√	√	√	√	
29	N947-2	947.14	947.31	4	1				19.51		√	√	√	√	√	√	novo-LNP I
30	N1150-1	1150.12	1150.39	4	2				21.01		√	√	√	√	√	√	LNnH

^1^F, fucosylated PMOs; N, neutral core-structured PMOs; S, sialylated PMOs; SF, fucosylated and sialylated PMOs. ^2^Expt, experimental m/z values; cal, calculated m/z values; Hex, hexose; Fuc, fucose; HexNAc, N-acetylhexosamine; NeuAc, N-acetylneuraminic acid; NeuGc, N-glycolylneuraminic acid. ^3^Proposed structures are presented according to Oxford symbol nomenclature: Blue circles and yellow circles indicate glucose (Glc) and galactose (Gal), respectively; blue squares represent N-acetylglucosamine (GlcNAc); purple and brillant blue diamonds represent N-acetylneuraminic acid (NeuAc) and N-glycolylneuraminic acid (NeuGc), respectively; red triangles represent fucose (Fuc). ^4^structure names given in other articles to PMOs, BMOs or HMOs. *Proposed structures need to be confirmed further.

**Table 2:** Compositions and proposed structures of Porcine milk oligosaccharides (PMO) identified in this study----Unknown Structures

PMO of unknown structures																	
No.	Designation^1^	m/z^2^ (expt)	m/z^2^ (cal)	Hex	HexNAc	Fuc	NeuAc	NeuGc	Retention Time (min)	Proposed Structures^3^	Colostrum		Transitional Milk		Mature Milk		Notes^4^
											Gilt	Sow	Gilt	Sow	Gilt	Sow	
**Acidic PMO**																	
1	S752-2	752.22	752.25	1	1		1		12.97		√	√	√	√	√	√	
2	S752-4	752.22	752.25	1	1		1		17.73		√	√	√	√	√	√	
3	S914-1	914.15	914.29	2	1		1		13.40		√	√	√	√	√	√	
4	S914-2	914.15	914.29	2	1		1		14.23		√	√	√	√	√	√	
5	S955-1	955.25	955.33	1	2		1		12.90		√	√	√	√	√	√	
6	S955-2	955.25	955.33	1	2		1		14.51		√	√	—	√	√	√	
7	S955-3	955.25	955.33	1	2		1		17.83		√	√	√	√	—	√	
8	S1076-1	1076.12	1076.35	3	1		1		15.16		√	√	√	√	√	√	
9	S1279-1	1279.06	1279.43	3	2		1		17.04		√	√	√	√	√	√	
10	S1367-1	1367.09	1367.45	3	1		2		17.56		√	√	√	√	√	√	
11	S1367-2	1367.09	1367.45	3	1		2		18.43		√	√	√	√	√	√	
12	S1441-1	1441.15	1441.48	4	2		1		19.91		√	√	√	√	—	√	
13	S1441-2	1441.15	1441.48	4	2		1		20.82		√	√	√	√	√	√	
14	S1570-1	1570.13	1570.53	3	2		2		18.14		√	√	√	√	—	—	
15	S1570-2	1570.13	1570.53	3	2		2		19.13		√	√	—	√	—	—	
16	S1732-2	1732.11	1732.58	4	2		2		22.67		√	√	√	√	—	√	DS-LNnH
17	S1732-3	1732.11	1732.58	4	2		2		23.89		√	√	√	√	—	—	DS-LNnH
**Neutral PMO**																	
18	N664-1	664.16	664.23	1	2				11.47		√	√	√	√	√	√	
19	N664-2	664.16	664.23	1	2				11.66		√	√	√	√	√	√	
20	N664-3	664.16	664.23	1	2				12.83		√	√	—	√	—	—	
21	N664-4	664.16	664.23	1	2				16.84		√	√	—	—	—	—	
22	N785-2	785.15	785.26	3	1				22.07		√	√	√	√	—	—	
23	N988-1	988.16	988.34	3	2				17.44		√	√	√	√	√	√	
24	N988-2	988.16	988.34	3	2				19.13		√	√	√	√	√	√	
25	N1150-2	1150.12	1150.39	4	2				22.53		√	√	√	√	√	√	

^1^F, fucosylated PMOs; N, neutral core-structured PMOs; S, sialylated PMOs; SF, fucosylated and sialylated PMOs. ^2^expt, experimental m/z values; cal, calculated m/z values; Hex, hexose; Fuc, fucose; HexNAc, N-acetylhexosamine; NeuAc, N-acetylneuraminic acid; NeuGc, N-glycolylneuraminic acid.^3^Proposed structures are presented according to Oxford symbol nomenclature: Blue circles and yellow circles indicate glucose (Glc) and galactose (Gal), respectively; blue squares represent N-acetylglucosamine (GlcNAc); purple and brillant blue diamonds represent N-acetylneuraminic acid (NeuAc) and N-glycolylneuraminic acid (NeuGc), respectively; red triangles represent fucose (Fuc). ^4^structure names given in other articles to PMOs, BMOs or HMOs.

### Expression of neutral, fucosylated and sialylated PMOs during lactation

**Figure 2:**
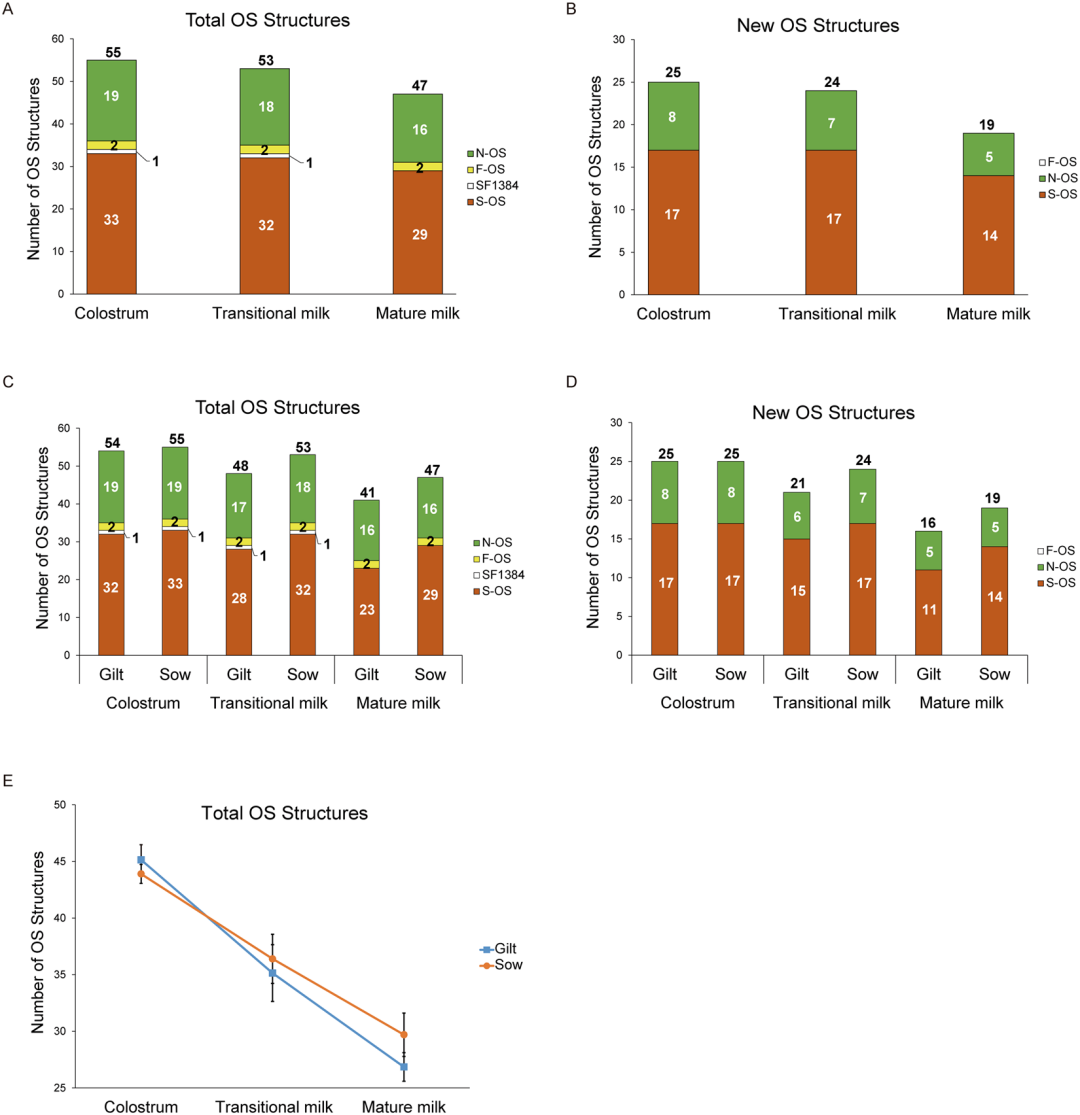
Diversity in the total porcine milk oligosaccharide (PMOs) structures during lactation. (**A**) Total milk oligosaccharide structures; (**B**) New milk oligosaccharide structures; (**C**) Total milk oligosaccharide structures in gilt *vs* sow; (**D**) New milk oligosaccharide structures in gilts *vs* sow; (**E**) Total milk oligosaccharide structures in gilt *vs* sow through lactation. Fucosylated (F-OS), neutral core (N-OS) and sialylated (S-OS) milk oligosaccharides.

The chemical structures and abundance of neutral, fucosylated and sialylated PMOs during lactation were characterized from 10 lactating sows and 7 gilts by mass spectrometry and summarised in Tables 1 and 2. Of the 55 PMO structures identified in this study, 34 were sialylated and 21 were neutral (Tables 1, 2). The neutral sugars included the two fucosylated structures, F566-1 and F566-2, but excluded the sialylated-fucosyl structure SF1384 (Table 1). Importantly, a total of 25 new (17 sialylated and 8 neutral) PMO structures that have not been identified previously were reported (Table 2)^14,32,33^, based on their mass spectral profiles (Fig. 1A–D),

In porcine milk, sialylated oligosaccharides were the predominant structures, comprising 34 of the total of 55 PMOs and new PMOs (Fig. 2A,B). This finding shows that the expression profile of sialylated PMOs is more similar to BMOs than to HMOs. In contrast, HMOs are also highly fucosylated, accounting for up to 70% of the MO species^34^. Previously, 19 fucosylated PMOs were identified in porcine milk^14,31,32,35–37^. However, in our study, only 3 known fucosylated structures were detected (Table 1, Fig. 2A,C), due to different breeds of swine, stage of milk collection, parity of swine milk and methodology used for MO analysis.

### Changes in the structural diversity of the PMOs in gilts and sows during lactation

An unresolved question that has not been adequately addressed is how the chemical structures of PMOs may change during the course of lactation and whether the structures of PMOs change as a function of different parities. Therefore, the structural diversity of PMOs in the milk of female pigs which had been bred at least once (sow) with those which had been bred for the first time (gilt) were assessed in colostrum (day 1), transitional milk (day 3) and mature milk (day 15–21). A comparison of the total number of PMO structures expressed during the course of lactation in gilt and sow milk is shown in Fig. 2C,D. The total number of structures in colostrum was similar between sow (55) and gilt (54) milk respectively. The number of sialylated oligosaccharides in colostrum showed little variation between sow and gilt (33 and 32 structures, respectively), as the one known sialylated PMO 3′/6′-SLNP, (S1238-2) in gilt milk was not detected (Table 3). While the number of new PMO structures in colostrum was identical in both sow and gilt milk (Fig. 2D). A decrease in the total number of PMO structures over the course of lactation was evidenced in both sow and gilt milk. However, sow milk showed only a slight decrease relative to the gilt milk in transitional milk (53 *vs*. 48 structures) and in mature milk (47 *vs*. 41 structures) (Fig. 2C): however, differences were not statistically significant when considered over all animals (Fig. 2E). In transitional milk, there were more sialylated structures (32) in sow milk compared with gilt milk (28 structures). Besides fucosylated MOs, the sow and gilt milk contained a similar number of neutral MO (18 *vs*. 17) structures in transitional milk (Fig. 2C). In mature milk, the number of sialylated structures was also greater in sow than in gilt milk (29 *vs*. 23 structures), while the number of neutral MOs was identical (16 structures) in total PMO (Fig. 2C). On the basis of these findings we conclude: (1) In colostrum, there was essentially no difference in the total number of neutral (19 each), sialylated (33 *vs*. 32) and fucosylated (2 *vs* 2) MOs between the sow and gilt; (2) The major changes in different numbers of MO structures between sow and gilt milk were found in transitional milk (53 *vs*. 48). Interestingly, sialylated MO structures were responsible for most of these differences (28 *vs*. 32), while there was essentially no change in the number of neutral PMOs (from 17 to 18) in transitional milk; (3). The total number of PMO structures in mature milk increased with parity from gilt to sow (41 *vs*. 47), with the sialylated PMO known structures of S1441-3, S1043, SF1384 and unknown structures of S1570-1, S1570-2, S1732-3 not being present in gilt milk. There was, however, no change in the number of neutral PMO in gilt and sow milk (16 structures each).

**Table 3:** PMO structures present in each lactation stage in sow and gilt.

	Isomer	Structure		Isomer	Structure
OS present only in colostrum, but not in transitional and mature milk	*N664-4*		OS present only in sow milk, but not in gilt milk at the stage of transitional milk	*N664-3*	
OS present in colostrum and transitional milk, but not in mature milk	*N664-3*			*S955-2*	
	*N785-2*			*S1570-2*	
	SF1384			S1043	
	S1441-3		OS present only in sow milk, but not in gilt milk at the stage of mature milk	*S955-3*	
	*S1570-1*			S1043	
	*S1570-2*			*S1441-1*	
	*S1732-3*			S1732-1	
OS present only in sow milk, but not in gilt milk throughout the whole lactation stage	S1238-2			*S1732-2*	

Italic represents new structures.

As shown in Fig. 2D, there was a lactation-stage related and progressive decrease in the total number of new PMO structures by 36% and 24% in sow and gilt milk respectively over the course of lactation. For example, the total number of new PMO structures decreased from 25 in colostrum of both sow and gilt milk to 24 and 21 in transitional milk and then further decreased to 19 and 16 in mature milk respectively. Similarly, both the number of new sialylated and neutral PMO in mature milk from both the sow and gilt showed a lactation-stage related decrease from colostrum (Fig. 2D). Interestingly, there were no changes in the number of new sialylated PMO structures from colostrum to transitional milk in sow milk. In gilt milk, however, this number decreased from 17 in colostrum to 15 in transitional milk (Fig. 2D). In mature milk, 14 and 11 new sialylated structures were detected in gilt and sow respectively. There were no differences in the number of neutral oligosaccharide structures in mature milk between sow and gilt (5 structures each, Fig. 2D).

Over the course of lactation from colostrum through transitional to mature milk, several interesting and specific structural changes in the sialylated and neutral oligosaccharides are noteworthy as summarized in Table 3. The unknown neutral PMO structure, N664-4, could be detected only in colostrum, but not in transitional and mature milk in both parities. Seven structures including known 2 (SF1384, S1441-3) and unknown 5 (N664-3, N785-2, S1570-1, S1570-2, S1732-3) could be detected in both colostrum and transitional milk, but not in mature milk. Interestingly, the known sialylated PMO, S1238-2, was only detected in sow milk, but not in gilt milk throughout lactation. The number of known neutral PMO structures, including fucosylated PMO structures, did not change throughout the course of lactation. Thus, the overall changes in PMO structural number were complex, MO species specific, parity dependent and lactation stage specific.

All known PMO species are derived from two basic core disaccharide structures, lactose and LacNAc. Lac-core oligosaccharides usually represent the majority of structures considering both their number and diversity. In our study, we identified a total of 55 PMO structures in colostrum, including 38 based on the Lac-core structure and 17 based on LacNAc (Supplementary Fig. 1 and Tables 1 and 2). During lactation, the number of Lac-core structures decreased to 35 in transitional milk and 30 in mature milk, while the number of LacNAc based structures remained relatively constant during lactation (Supplementary Fig. 1, Supplementary Table 1).

### Quantification of the level of PMOs over the course of lactation in gilts and sows

**Figure 3:**
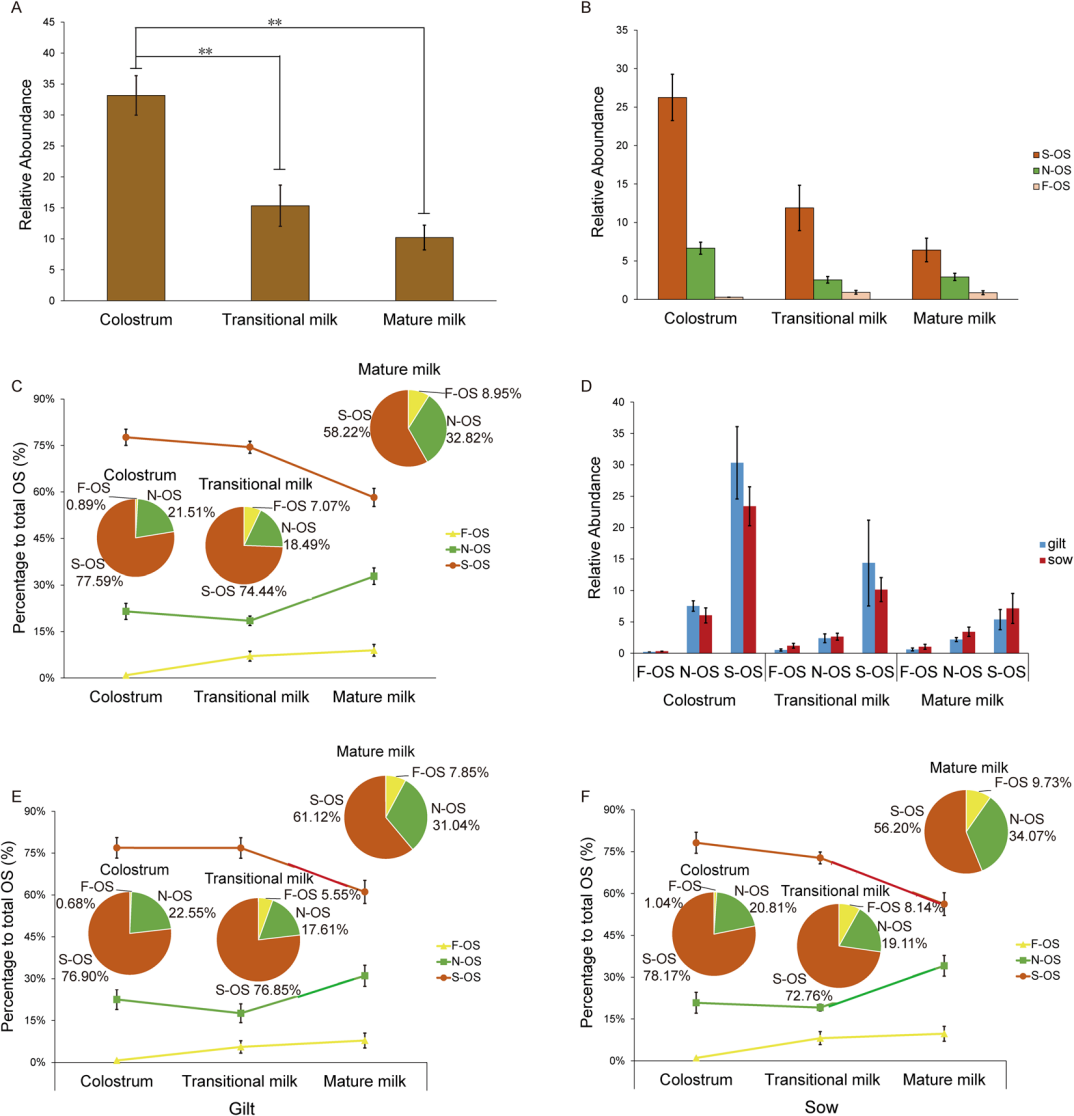
Change in abundance of PMOs during lactation. (**A**) Relative abundance of fucosylated-MOs, neutral-MOs and sialylated-MOs; (**B**) Relative percentages of fucosylated-MOs, neutral-MOs and sialylated-MOs to total PMOs; and (**C**) Changes in the relative abundance of fucosylated-MOs, neutral-MOs and sialylated-MOs over lactation; (**D**) Relative percentages of each class to total abundance of PMOs; and (**E**) Changes in expression in gilts and sows at different stages of lactation. Asterisk represents statistical significance, P < 0.05; Double asterisk represents high statistical significance, P < 0.01.

To determine how the level of PMOs may change across lactation in gilt and sow milk, the relative abundance of PMOs in colostrum, transitional and mature milk was quantitatively analyzed using HPLC-ESI MS/MS, as described under “Methods”. As shown in Fig. 3A, the relative abundance of the total level of PMOs was *ca*. 33, 15 and 10 (peak area ratio to internal reference) in colostrum, transitional milk and mature milk, respectively. The top 5 concentrations of each of the PMO isomers were summarised in Table 4. Within the same stage of lactation, for example in colostrum, the difference in the relative concentration between the highest level of the sialylated oligosaccharides, represented by S711-1 in gilt (44.51% to total OS) (Table 4), and the lowest, represented by the 6′-NeuGcLacNac oligosaccharide S768 (0.00092% to total OS), was greater than 40,000-fold. Consistent with previous observations^14^, the total relative abundance of the PMOs showed a significant decrease over the course of the three stages of lactation (Fig. 3A). The notable decrease in the levels of both the sialylated and neutral oligosaccharides during lactation is shown in Fig. 3B. Interestingly, the fucosylated oligosaccharides showed an increase in expression levels in the transitional from colostrum to mature milk (Fig. 3B). The percentage of the total fucosylated oligosaccharides increased from 0.89% in colostrum to 7.07% in transitional milk, and to 8.95% in mature milk (Fig. 3C). This is an important finding because it shows an up-regulation in the level of fucosylated oligosaccharides in porcine milk during lactation, a finding that confirmed the recent report of J. Salcedo^38^. During the same lactation stage, the level of sialylated oligosaccharides remained relatively constant from colostrum (77.6%) to transitional milk (74.4%), but decreased to 56.2% in mature milk. This change was consistent with the increase in the level of neutral oligosaccharides from colostrum (21.5%) and transitional milk (18.5%) to mature milk (32.8%) (Fig. 3C).

**Table 4:** Isomers top 5 in porcine colostrum, transitional and mature milk.

Isomer (%) top 5 in amount	1	2	3	4	5	
Colostrum	Gilt	**S711-1** (44.51%)	**S914-1** (22.50%)	**N582-1** (13.26%)	**N785-1** (3.28%)	**N1150-2** (1.43%)
	Sow	**S711-1** (53.28%)	**S914-1** (16.01%)	**N582-1** (12.05%)	**N785-1** (2.87%)	**S711-2** (1.67%)
Transitional milk	Gilt	**S711-1** (63.73%)	**N582-1** (9.30%)	**S914-1** (7.10%)	**F566-1** (5.20%)	**N785-1** (3.63%)
	Sow	**S711-1** (54.34%)	**S914-1** (9.59%)	**N582-1** (8.59%)	**F566-1** (7.76%)	**N785-1** (3.80%)
Mature milk	Gilt	**S711-1** (43.31%)	**N582-1** (14.34%)	**F566-1** (7.57%)	**N785-1** (7.21%)	**S914-1** (6.87%)
	Sow	**S711-1** (36.83%)	**N582-1** (16.80%)	**S914-1** (10.02%)	**F566-1** (9.09%)	**N785-1** (7.21%)
F566-1: 2′-FL	N582-1: Isogiobiotriose/3′/6′-GL	N785-1	N1150-2	S711-1: 3′-SL;	S711-2: 6′-SL;	S914-1
						

Comparison of the relative abundance of sialylated, neutral and fucosylated PMOs in gilt and sow during lactation showed that sialylated and neutral PMO declined during lactation and fucosylated PMO increased during lactation (Fig. 3D). Sialylated MO was the predominant species in sows (56.2%~78.17%) and gilts (61.12%~76.90), followed by neutral MO (17.61~34.07%) and then fucosylated MO (0.68%~9.73%) (Fig. 3E, F). By 20 days (mature milk), only about 18.3% and 36.1% of the initial content of sialylated MOs was present in sow and gilt milk. In neutral MOs, however, 32.9% and 82.4% of the initial content remained in sow and gilt milk respectively; thus the sialylated MOs were the most variable components of the PMOs. The difference in abundance of sialylated, neutral and fucosylated PMOs within the same stage of lactation was not statistically significant between sow and gilt (P > 0.05), however a significant difference was evidenced in one of each isomer between sow and gilt within same stage of lactation, in colostrum and transitional milk (P < 0.05, Supplementary Fig. 2).

### Specific structural variation in porcine oligosaccharides during lactation

**Figure 4:**
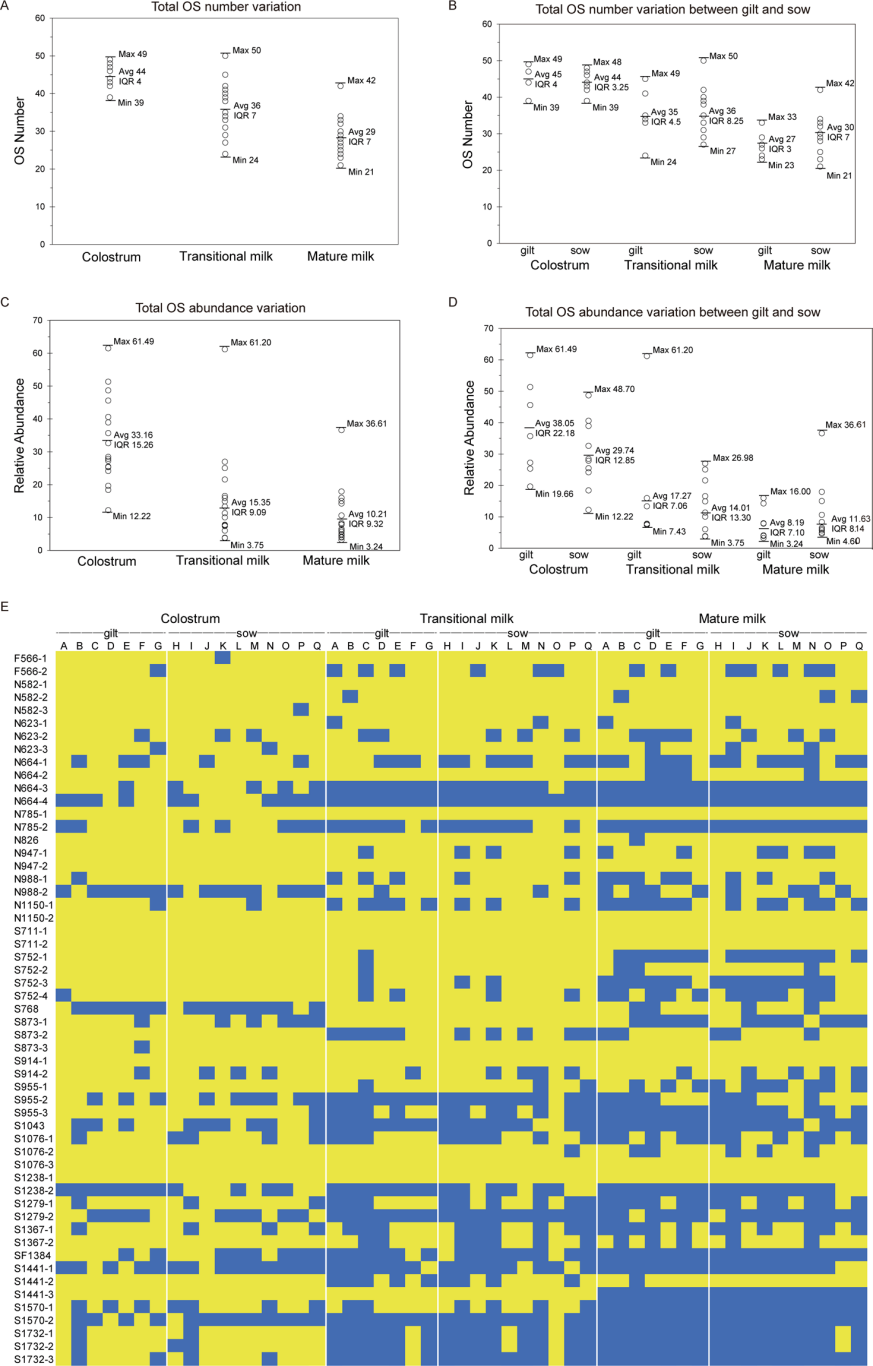
Variation in PMO structures in different milk samples through lactation. (**A**) Structural diversity of PMO in different stages of lactation, the ball represented each milk sample in (B,C,D panel); (**B**) Structural diversity of PMO between gilt and sow throughout lactation. (**C**) Variation in relative abundance of total PMOs during lactation; (**D**) Variation in relative abundance of total PMOs between gilt and sow during lactation. Horizontal bars with figures indicate the maximum, mean and minimum number of PMO (**A** and **B**); or relative abundance (**C** and **D**) in samples from different pigs and their Inter-Quartile Range (IQR) number. (**E**) Detectable isomers in each sample are presented as the heat map. The horizontal letter, “A to Z”, on top represents each milk sample and the vertical m/z, “F566-1 to S1732-3”, indicates individual isomers in each sample. Structures present in a sample were indicated with yellow colour, and if not, it was indicated with blue colour.

The structure of PMOs showed a significant variation in different milk samples. For example, the 55 isomers of PMOs identified in this study were not present in all milk samples tested, showing individual variation across sows/gilts. In fact, the average number of PMO structures identified in colostrum was 44, while the maximal number identified in certain porcine milk samples reached up to 49 (Fig. 4A). This degree of variation was represented as the Inter-Quartile Range (IQR)^39^. The IQR of PMO structural numbers were 4, 7 and 7 in colostrum, transitional and mature milk, respectively, showing that the diversity of structural numbers in PMOs species increased during lactation (Fig. 4A). Our results demonstrated that there were differences between gilt and sow milks in the OS structural numbers and abundance. In colostrum, the variation in relative abundance and diversity was greater in the gilt than the sow (Fig. 4B,D), however across lactation, the MO structural number, abundance and diversity were greater in the sow than in the gilt. This finding indicates that the PMO population remains more stable in the sow than in the gilt. Moreover, the variation in the total abundance of oligosaccharides in different milk samples also showed a large variation. For example, the relative abundance of the oligosaccharides in colostrum ranged from 12.22 to 61.49, with an IQR of 15.26 (Fig. 4C). The IQR in the level of oligosaccharide abundance in the transitional from colostrum to mature milk, decreased from 15.26 to 9.32, with transitional milk at 9.09. This finding indicates that the PMO content varies greatly during lactation, both within and between individuals, more than any other component of porcine milk (Fig. 4C). There were only 5 sialylated oligosaccharide isomers present in all milk samples throughout lactation, including, S711-1 (3′-SL), S711-2 (6′SL), S914-1, S1076-3 (3′/6′-SLNnT), S1238-1 (3′/6′-SLNP 1), and 4 neutral oligosaccharides, N-582-1 (Isoglobotriose/3′/6′-GL), N785-1, N947-2 (novo-LNP 1), N1150-2 (Fig. 4E). As shown in Table 4, most of these structures are major components in colostrum (Table 4).

## Discussion

The evolution of animal species has relied heavily on breast milk and its nutritional composition. Accordingly, diversity in the chemical structure and concentration of milk oligosaccharides may be related to animal evolution, benefit to protection of new-borns and successful reproductive outcomes. An important outcome of our present studies is a comprehensive and comparative analysis of MOs from 10 sows and 7 gilts at three different stages of lactation. These porcine milk samples were labelled with the aniline isotope for identifying oligosaccharides via mass spectroscopy. A similar experimental method was applied in studies of MOs from human and cow^40^. However, this is the first study using the aniline stable isotope labelling strategy to analyse the expression profile of PMOs through the stages of lactation. Seventeen of the new structures sialylated and 8 neutral oligosaccharides were identified in this study. We also identified 30 previously described oligosaccharide structures, of which 17 were sialylated and 13 were neutral. A total of 3 known fucosylated structures were identified, one of which was also sialylated (SF 1384), yet no new fucosylated structures were discovered in porcine milk at any stage of lactation. Only one of the five previously described sialylated Neu5Gc, S768 in both sow and gilt milk was detected in our study (Fig. 4E). Interestingly, out of 17 milk samples, this moiety was identified only in 3 colostrum and 5 mature milk samples. While it was identified in all 17 transitional milk samples collected from both sows and gilts. Therefore, sialylated Neu5Gc MOs are expressed routinely in porcine milk throughout lactation regardless of parity.

The total number of PMOs showed a significant decrease over the three stages of lactation (Fig. 2A–C), of which the decrease observed in gilt milk (24%)was larger than in sow milk (15%) from colostrum and mature milk. Interestingly, in both gilt and sow milk the decrease in the rate of expression of neutral PMO was the same (by 16% from colostrum to mature milk). In sialylated PMO however, the total number of structures in gilt milk decreased 2 times faster from colostrum to mature milk than for the sow milk (28% in gilt *vs* 12% in sow). Compared to sow milk, the known structures of S1043 (DSLN), S1238-2 (3′/6′-SLNP I), S1732-1 (DSLNnH) and unknown structures of S955-3, S1441-1, S1732-2 could not be detected in gilt mature milk. The total number of fucosylated structures of PMO remained the same in both gilt and sow milk throughout lactation. In newly identified structures of PMO, the total number of neutral PMO showed the same rate of decrease between the gilt and sow, while sialylated PMO in gilts decreased twice as fast as in the sow over the 3 weeks lactation used in this study. However, the rate of decrease in the expression of new structures for neutral and sialylated MOs was about 1.3~2 fold faster than for the known PMO structures over the course of lactation. Elucidation of the differences in PMO structures found in sow and gilt milk may assist in improving our understanding of the mechanisms responsible for lower milk production, and the delivery of lower birth weight piglets from gilts relative to sows^41^.

With the inclusion of the 25 new structures identified in the current study, a total of 119 MOs (previous reported 94) oligosaccharides have been characterized from porcine milk. Collectively, these results, principally derived from mass spectroscopy, have the potential to provide a greater understanding of the critical role of different concentrations of MO in improving health and disease prevention. Furthermore, sialylated Neu5Gc MOs are not expressed in human milk throughout lactation regardless of parity. Thus, when these findings are coupled with the outcome of contemporary studies relating human MOs with the functionality of enteric microbiota^42^, we may be better able to identify the milk oligosaccharides required to improve the efficacy of infant formulae.

The total concentration of PMO was highest in colostrum and declined during the course of lactation. The importance of colostrum on growth and development in human infants has been well documented^43^. Sialylated PMOs accounted for 58.2–77.6% of the total PMOs over the course of lactation (Fig. 3A–C). The comparative abundance of gilt and sow milks is shown in Fig. 3E and F. The high levels of expression of the sialylated MOs are suggestive of a fundamental nutritional role in biochemical and physiological processes for optimizing neurodevelopment, immune function and growth and development of newborns. Compared to the proportion of 10~30% of sialylated oligosaccharide in human milk, porcine milk contained a proportionally higher percentage (58~78%) of sialylated oligosaccharides in total MOs, although the absolute amount of sialylated oligosaccharides in PMO is much lower than that in HMO. Accordingly, the results of our study on the abundance and diversity of PMOs provides further evidence to support the importance of the nutritional role of milk oligosaccharides across species.

Previous studies have shown that fucosylated PMO accounted for about 1–26% of the total OS in abundance (Table 5). Interestingly, overall fucosylated PMOs accounted for < .∼1% of the total oligosaccharides in colostrum, increasing to about 9% in mature milk (Fig. 3C). However, the two isomers of fucosylated PMOs were expressed in different amounts. 2′-FL (F566-1) was up-regulated, but 3′-FL (F566-2) was down-regulated (Supplementary Figs 3 and Fig. 2). This is an important finding because it shows that the synthesis of fucosylated PMOs oligosaccharides is upregulated overall during lactation before^37,38^, the individual isomer of fucosylated PMOs, 3′-FL was decreased during lactation. To our knowledge, this is the first report clarifying a lactation-stage related contra-indicatory regulation of different fucosylated oligosaccharide synthesis in porcine milk, although such an increase of 2′-FL over the course of lactation has been observed in human milk^44^.

**Table 5:** Comparison of PMO with BMO and HMO.

	BMO		PMO (previous study)		PMO (this study)		HMO	
	Structure numbers (n)	Percentage (%)	Structure numbers (n)	Percentage (%)	Structure numbers (n)	Percentage (%)	Structure numbers (n)	Percentage (%)
Total number of OS identified	100		94		55		>200	
Sialylated OS	35	50–90	43	16–80	34	58–78	>55	10–30
Neu5Gc-containing OS	6		5		1		—	
Phosphorylated OS	4		1		—		—	
Neutral OS (excluding Fuc-OS)	47	10–50	37	20–80	19	21–32	>10	<10
Fucoslylated OS	16	<1	19	1–26	3	1–9	>93	50–70
Both Sialylated and Fucoslyated OS	—		6		1		>35	
Common OS structures with HMO	15		25		14			

To our knowledge, the structures of 94 porcine^14,32,33,35–38^, 100 bovine^9,10,22,32^, and >200 human milk oligosaccharides^7,8,11,20^ have been reported. Comparatively, there are several striking differences in the structures and percentages of the total number of oligosaccharide structures in human, porcine and bovine milk, as summarized in Table 5. These include the following: (1) a significantly larger number of fucosylated oligosaccharides, >93 was reported in human milk, which accounts for about 70% of the total amount of HMOs^7,8,11,20^; (2) in contrast to human milk, there was a significantly lower number of fucosylated structures in cow (16 structures) and pig (19 structures) milks, which account for <1% of the total amount of MOs in the cow^9,10,22,32^, and 1–26% in the pig^35–38^. (3) There were >55 sialylated structures identified in human milk^8,11^ and 35 sialylated structures were reported in bovine milk^9,10,32^ and the 43 identified in pig milk^14,32,33,35,36^. Notably, the number of sialylated structures accounted for 50–90% of the total structures in cow and 16–80% of the structures in pig milk; (4). Four phosphorylated oligosaccharides have been identified in bovine milk^10,22^, and one in pig milk^36^. No such phosphorylated oligosaccharides have been reported in human milk; (5). Six isomers of both sialylated and fucosylated oligosaccharides have been reported in porcine milk, and one was identified in this study (SF1384 in Table 1). Such isomeric forms are a relative common structural feature of HMOs, with >35 having been identified^7,8,11^. However, no such isomers have been reported from BMOs (Table 5). There were 25 common MO structures present in porcine and human milk^14,32,33^, while only 15 BMOs are present in human milk^9,10,22,32^. Therefore, the composition of PMOs may more closely resemble that of HMOs, suggesting that porcine milk is a better “transitional” milk than bovine milk for studies linking milk nutrition to human infant health and development.

A final interesting and enigmatic perspective coming from our study is that a known structure of α-2,8-ketosidic linkages disialylated MO, Neu5Acα2,8Neu5Acα2,3Galβ1,4GlcNAc is found in both sow and gilt milk over the course of lactation. This disialylated MO seemly is one of most highly bioactive MO compared to sialyllactose in human milk^45,46^. In comparison to monosialylated MO, disialylated MO protects neonatal rats from necrotizing enterocolitis^46^. However, such a glycan linkage has not been well documented nor confirmed in structural studies of milk oligosaccharides. Whether this finding may relate to an enhanced lability of α-2,8-ketosidic linkages during MS/MS analysis, is currently under study in our laboratory. It seems less likely that the mammary gland lacks members of the ST8SiaI-VI sialyltransferses family, at least based on the expression of the α-2,8-polysialic acid glycan in some human breast cancers^47^.

Previous studies have shown that there were no significant differences in the concentrations of lactose, protein and fat between gilt and sow milk over lactation^48^ and porcine colostrum yield was not affected by age, body weight, duration of parturition or rectal temperatures Devillers *et al*.^49^. However, there was a difference in colostrum yield between sows of different parity with the highest production being found in the second and third parity^50^. Gilts are more susceptible to deliver low birth weight piglets than sows in pig production^41^, and therefore the unique abundance and structural diversity of gilt MO may be important to meet the high nutritional requirement of their offspring to support their fast growth and development during early life. In pig production gilts are joined as soon as they attain puberty, by which time they have still not have attained a mature body weight. In managing this process, the gilt may well find that the provision of higher concentrations of the MO to their litter may help compensate for deficiencies in milk volume and therefore total nutrient availability.

## Methods

The experimental protocol was performed in accordance with guidelines established by the National Health and Medical Research Council of Australia and National Natural Science Foundation of China. Our study protocol was approved by The Animal Care and Ethics Committee of Charles Sturt University, Wagga Wagga, NSW, Australia (13/103).

### Animals

Sows (n = 10) and gilts (n = 7) of a Landrace, Belgian Landrace, Large White and Duroc cross-breed (*Sus scrofa*) from the Pig Improvement Company (PIC) facility at Grong Grong, N.S.W, Australia were the source for collection of all milk samples. The sows and gilts were healthy and aged 1~4 yr with uncomplicated perinatal histories. The mean gestational age of sows and gilts were *ca*. 114 days.

Pigs were fed a commercial gestation diet of *ca*. 2.4 kg/day from mating to the end of gestation, and then a lactation diet of *ca*. 5.8 kg/day for the 21 days to weaning. All animals had access to water *ad libitum*. Animals were housed under standard commercial conditions.

### Milk sample collection from gilt and sow

The details of milk sample collection from sow and gilts was the same as our published method^51^. Milk samples were collected from gilts and sows on colostrum at day 1, transitional milk at day 3 and mature milk at day 15 of lactation by manual expression during the same time period each morning (0900–1200 h). Unlike dairy cows, the porcine mammary gland does not have cisternae for storage of milk. Milk collection therefore can only be carried out after inducing the milk ejection reflex through release of oxytocin induced by the suckling stimulus of the piglets for at least one minute^52,53^. In this study, 2 to 5 mL of milk was collected from each gilt and sow after the milk let down was induced by the suckling stimulus from piglets during the 10- to 20-s window of opportunity. Samples were immediately placed on ice after collection and stored at −20 °C until analyzed.

### Sample Preparation of Porcine Milk Oligosaccharides (PMOs)

Preparation of porcine milk oligosaccharides PMOs was modified from the published procedure of Ninonuevo, M. R.^12^. Briefly, the frozen milk samples were thawed, mixed with an equivalent volume of ultrapure water, and centrifuged at 8000 × g for 30 min at 4 °C. After removal of the upper fat layer, 4 volumes of chloroform/methanol (2:1) were added to the sample and centrifuged as above. The supernatant fractions were transferred to fresh tubes and two volumes of absolute ethanol were added. After overnight incubation at 4 °C, the samples were centrifuged again as above and the supernatant fractions, enriched in the PMOs, were freeze-dried in a speed-vacuum unit (Savant ISS110, Thermo Fisher). The PMOs were further purified on 4.0 ml graphitized Carbon SPE columns (Carbograph Extract-Clean™, Altech, British) as described by Coppa *et al*.^1^. The column was washed with 3.0 ml water and eluted with the same volume of 25% acetonitrile (for neutral PMOs) and 25% acetonitrile containing 0.05% TFA (for acidic (sialylated) PMOs), respectively. The column eluates were combined and freeze dried with a speed-vacuum for further use.

### Derivatization of PMOs with *d*_0_-Aniline and *d*_5_-Aniline for HPLC-ESI MS/MS Analyses

Derivatisation of the PMOs was carried out using a modified published procedure of Jiang, K. *et al*.^31^. Briefly, the spin-dried PMOs samples were re-suspended in 100 μl ultrapure water, and 50 μl *d*_0_- or *d*_5_-aniline were added to the 100 µl samples followed by 50 μl of 1 M NaCNBH_3_ (Sigma Aldrich, St. Louis, MO, USA) in 30% acetic acid (*v*/*v*). The reaction mixtures were vortexed and sealed in a screw-capped tube. After incubation at 70°C for 20 min, the samples were then purified on a graphitized carbon SPE column (Carbograph Extract-Clean™ 4 ml column, Altech, British). The column was first washed with 15 ml ultrapure water, and eluted with 3 ml of 30% acetonitrile for the neutral PMOs and 3 ml of 30% acetonitrile containing 0.05% TFA for the sialylated (acidic) PMOs, respectively. Column eluates were dried in a speed-vacuum (Savant ISS110, Thermo Fisher) and dissolved in 50% acetonitrile for high-performance liquid chromatography-electrospray ionization mass spectrometry (HPLC-ESI MS) analysis.

### HPLC-ESI MS/MS Analysis of PMOs

Structural characterization and expression analysis of PMOs during lactation was carried out using our published HPLC-ESI MS/MS method with slight modifications^31^. For qualitative analysis, 15 μl of *d*_0_-aniline labeled PMOs were mixed with an equal volume of *d*_5_-aniline labeled PMOs, and the mixture was loaded for LC-MS/MS analysis. For quantitative analysis of PMOs, 25 μg/ml (final concentration) of β-cyclodextrin (Sigma Aldrich, St. Louis, MO, USA) was added to the *d*_0_-aniline-labeled PMOs as an internal MS standard, and 15 μl of this mixture was used for LC-MS/MS analysis. Analysis was carried out on a HPLC system (Thermo Fisher Scientific Inc, USA) with a XAmide (NH_2_) rapid resolution column (4.6 mm × 250 mm, 5 μm, Acchrom, China) linked to a linear ion trap ESI-MS system (Thermo Fisher Scientific Inc, LTQ Orbitrap XL, USA), and run in the positive-ion mode. The mobile phase was solvent A (acetonitrile), solvent B (water) and solvent C (ammonium acetate, 10 mM, pH 4.5) with a flow rate of 0.5 ml/min. The elution gradient was as follows: time = 0 min (t = 0), 75% A, 15% B, 10% C; t = 45, 55% A, 35% B, 10% C. The MS and MS/MS data were analyzed with Xcalibur software (Thermo Fisher Scientific Inc., USA). The PMOs were identified based on both the twin isotope peaks and their MS/MS fragments. Composition and sequences were analyzed and confirmed with GlycoWorkbench software^54^. The relative abundance of oligosaccharide in each sample was evaluated by comparing the peak area of each oligosaccharide component with that of β-cyclodextrin in extracted ion chromatography (EIC). The final concentration of all oligosaccharides was normalized to that of β-cyclodextrin.

### Statistical Analysis

The relative abundances of PMO were analyzed by a two-factor repeated measures analysis of variance model with 3 time points by using the Greenhouse-Geisser adjustment for asphericity. To investigate the different time trends for gilt and sow milk, the interaction between the dependent variable and grouping factor was determined. An overall comparison of relative abundances and different structures of PMOs between porcine milk of sow and gilt was obtained from the repeated measures analysis of the variance models, and comparisons at individual time points using t tests. All analyses were completed by SPSS for WINDOWS 19.0 (SPSS Inc, Chicago). Values were considered significant at P < 0.05.

## Electronic supplementary material

Supplementary Information
