# Supplementary material for: Characterization of porcine milk oligosaccharides over lactation between primiparous and multiparous female pigs
Source: Sci Rep. 2018 Mar 16;8:4688. doi: 10.1038/s41598-018-23025-x (PMC5856818; doi:10.1038/s41598-018-23025-x)
Supplement: Supplementary file 1 — Supplementary Information [file 41598_2018_23025_MOESM1_ESM.pdf]

Supplementary information for:

Characterization of porcine milk oligosaccharides over lactation between primiparous and multiparous female pigs

AUTHORS

[*1,2]Jinhua Wei, [*1,2]Zhuo A. Wang, [**1,3]Bing Wang, [3]Marefa Jahan, [4]Zhongfu Wang, [3]Peter C Wynn, [**1,2]Yuguang Du

AFFILIATIONS

1. State Key Laboratory of Biochemical Engineering, Institute of Process Engineering, Chinese Academy of Sciences, Beijing 100190, P.R. China.
2. Key Laboratory of Biopharmaceutical Production & Formulation Engineering, PLA, Beijing 100190, P.R. China.
3. Graham Centre for Agricultural Innovation, Charles Sturt University, Wagga Wagga, NSW, 2650, Australia
4. Key Laboratory of Resource Biology and Biotechnology in Western China, Ministry of Education, College of Life Sciences, Northwest University, Xi'an 710069, PR China

*Contributed equally

[**]Corresponding authors, emails:

B.Wang: biwang@csu.edu.au or Y.Du: ygdu@ipe.ac.cn;

Details for Structural characterization and quantification of PMOs by HPLC-ESI-MS/MS

Main text figure 1 is to show the details of identification of specific oligosaccharide structures. All twin isotope peaks in the spectra were further analyzed using the “GlycoWorkBench” software (version 2.1, https://code.google.com/archive/p/glycoworkbench/).

Structural analyses and annotation of the chemical structures of the PMOs were carried out as shown in Figure 1E by analysing the MS spectrum (top panel), HPLC chromatographic profile (middle panel) and the MS/MS spectrum (bottom panels) for each candidate PMOs peak observed in the mass spectra. For example, the MS spectrum and LC chromatographic profile for each PMOs peak, e.g. F566 (m/z 566.17), was examined as shown in the flow diagram (Fig. 1E). Isomers identified as twin isotope peaks were differentiated based on their retention times (Fig. 1E). The MS/MS profile for each isoform of the candidate PMOs peaks were then analyzed. Each sialic acid or neutral isomer was then annotated with the “GlycoWorkBench” software. The composition and proposed structures of the PMOs confirmed by both isotopic labelled twin peaks and MS/MS profile analyses during course of lactation are designated in Table 1 as either "known" or "unknown" PMO structures.

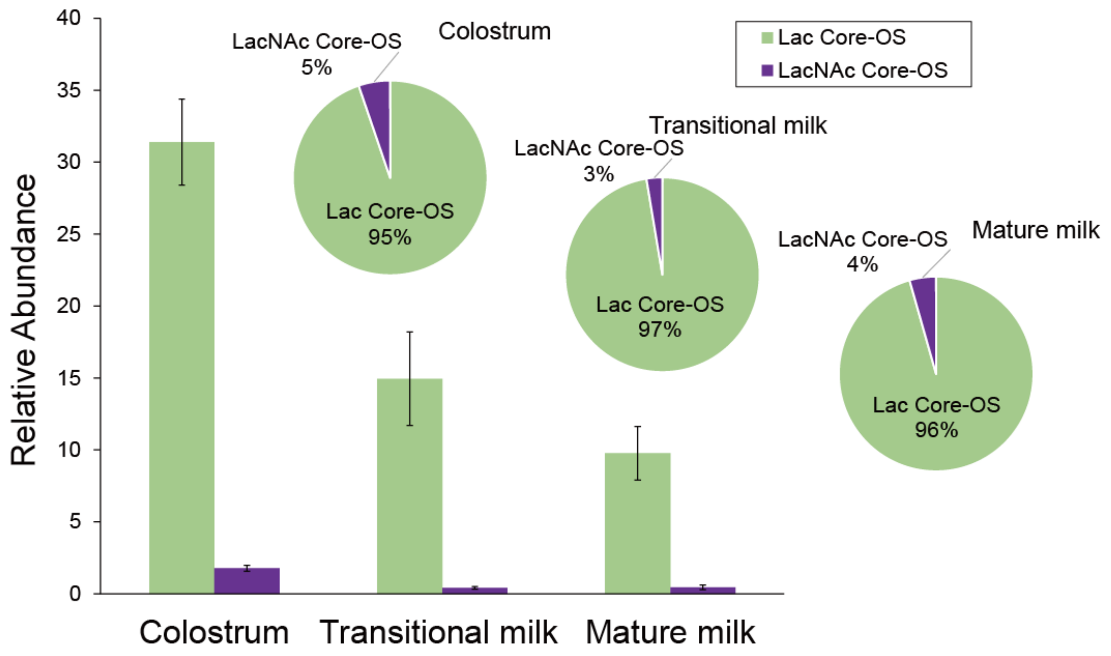

Figure S1. Change of Lac-Core- and LacNAc-Core PMOs during the developmental stages of lactation. Inserted triangular pie structures in the diagram show the ratio of both core PMOs in total PMOs during each stage of lactation.

Supplementary Table 1. Number of Lac Core- and LacNAc Core- OS structures through lactation

	Number of OS Structures		
	Colostrumm	Transitional milk	Mature milk
Lac Core-OS	38	35	30
LacNAc Core-OS	17	18	17

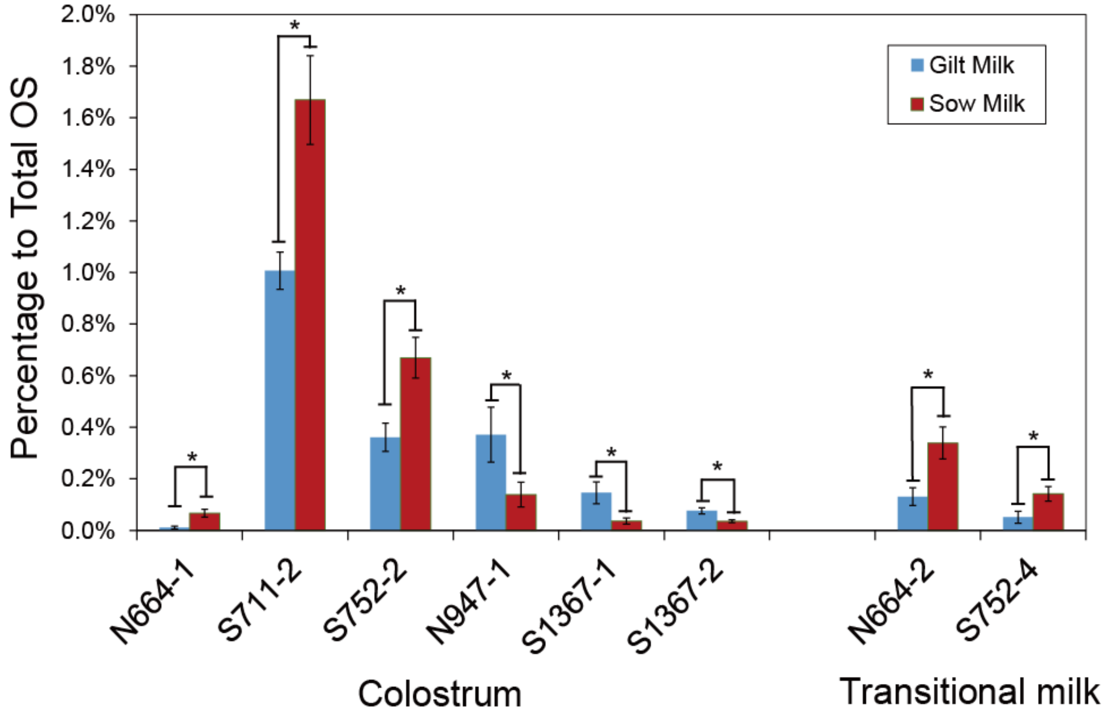

Figure S2. PMO structures with significantly different abundance between gilt and sow in samples of colostrum and transitional milk. Asterisk represents statistical significance, P<0.05.

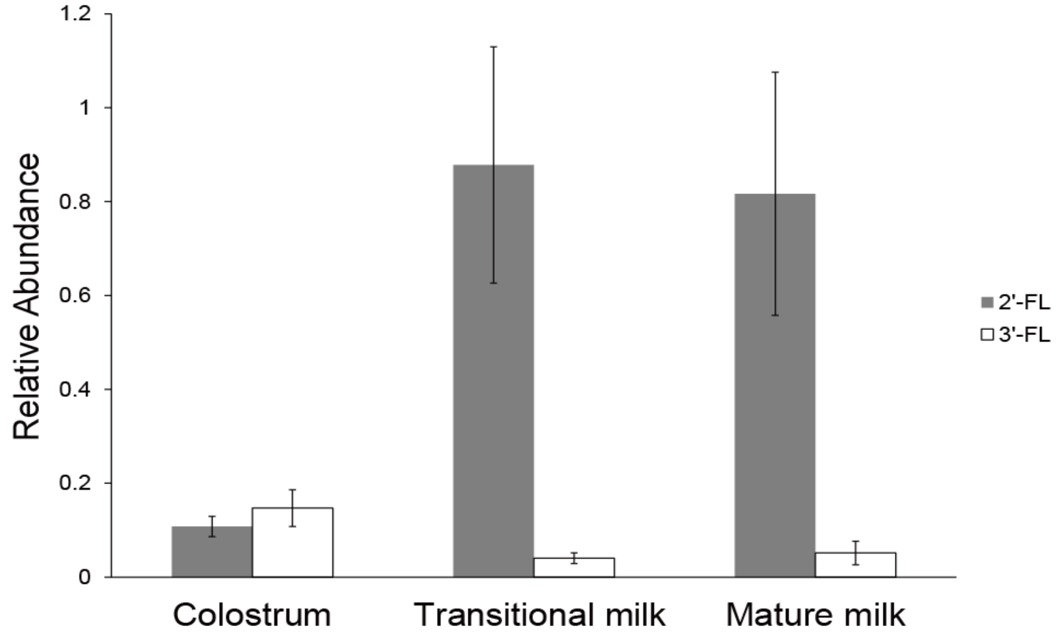

Figure S3. Change in relative abundance of 2’-FL (F566-1) and 3’-FL (F566-2) through lactation.

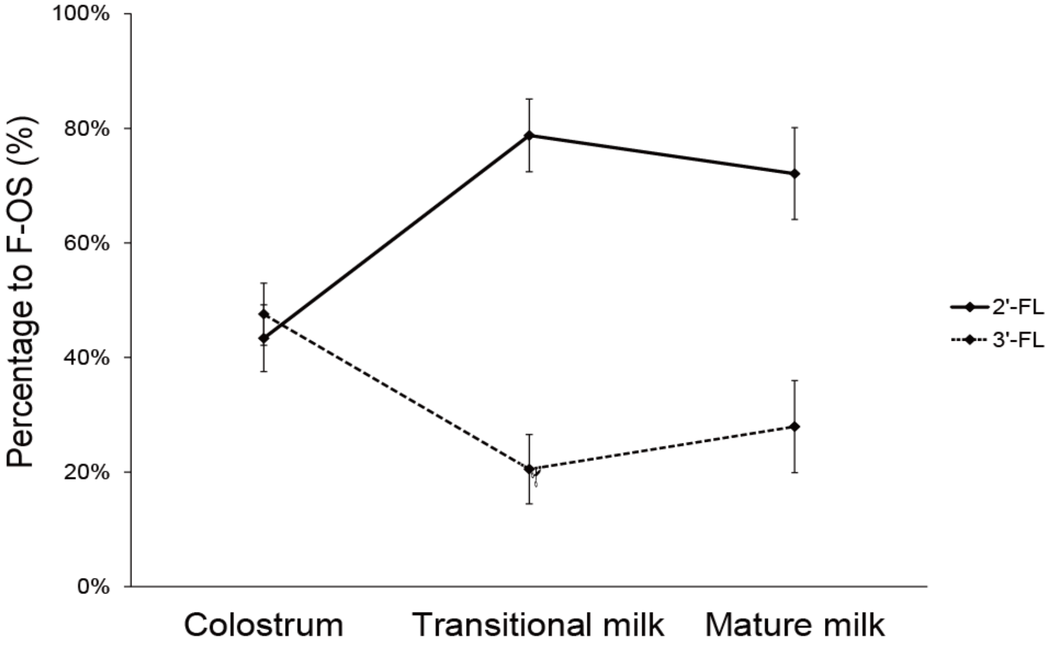

Figure S4. Change in percentage to total F-OS of 2’-FL (F566-1) and 3’-FL (F566-2) through lactation.
